# Capturing longer term surgical outcome measures as part of routine care of breast cancer patients

**DOI:** 10.1016/j.breast.2024.103758

**Published:** 2024-06-05

**Authors:** Astrid E. Leusink, Amy R. Godden, Nihal Yildirim, Antonia Randawa, Rebekah Law, Jennifer E. Rusby

**Affiliations:** a Institute of Cancer Research, Fulham Road, London, SW3 6JB, UK; b The Royal Marsden NHS Foundation Trust, Fulham Road, London, SW3 6JJ, UK; c The Royal Marsden NHS Foundation Trust, Downs Road, Sutton Surrey, SM2 5PT, UK

## Abstract

**Introduction:**

The transition away from routine clinical follow up after breast cancer towards imaging surveillance and patient-initiated contact limits opportunities for patients and doctors to communicate about the long-term effects of treatment. The ABS oncoplastic guidelines (2021) recommend that post-operative 2D images and patient-reported outcomes (PROMs) are routinely collected but give no guidance as to how best to implement this.

**Methods:**

From December 2019 until March 2024, women due for their year 3 or 5 surveillance mammogram at The Royal Marsden Sutton site were invited to complete a BREAST-Q questionnaire and attend medical photography. Panel assessment of photographs was undertaken. Results were presented to the oncoplastic MDT, including summary PROMs and illustrative case presentations. Free-text comments were shared with the relevant teams. Associations between demographic or clinic-pathological factors and uptake were investigated.

**Results:**

Of the 1211 women invited, 246 patients (20.3 %) completed BREAST-Q questionnaires, 182 (15.0 %) attended for medical photography and 114 (9.4 %) completed both. Uptake was not associated with age, ethnicity or surgical factors but patients with higher BMI were less likely to respond to the questionnaire. Patients who had undergone complex oncoplastic procedures were more likely to respond than those who had simple procedures. Patient-reported outcome results were in line with the published literature.

**Conclusion:**

Reviewing images with their paired PROMs and discussing free-text feedback was instructive for the team. Work is needed to identify barriers to patient participation and improve uptake to be representative of the overall patient population. Quantifying appearance in photographs would help summarise aesthetic outcome data.

## Highlights

• Study Highlights.
• Routine capture and processing of PROMs and aesthetic outcome data is important.
• There are numerous factors which may impact patient response rate.
• Breast units must be responsive to their local demographic and patients should be encouraged.
• Review of outcome data is useful for breast surgery teams.

## 1 Introduction

Breast cancer is the most common cancer affecting women, with 55 000 patients diagnosed every year in the UK [1]. Survival is excellent [2], and the quality of that survivorship therefore affects a large number of women for many years. Long-term patient satisfaction with aesthetic outcome is an important element of quality of life after breast cancer. Breast cancer patients have traditionally been followed up by their clinician on an annual basis for 5 years following the completion of their breast cancer treatment. This model of surveillance was recognised to be of little oncological benefit and found to be an anxiety-inducing experience for many patients [[3], [4], [5]]. The risk of local recurrence of breast cancer is low (1 % at 1 year and 5 % at 5 years) [6,7] and the majority of recurrences are identified by patients themselves, with clinical examination providing little value in identifying recurrent disease over and above annual mammography [7,8]. A study by Beltran-Bless et al. highlighted this, with only 1.14 % of local recurrences detected by healthcare providers at routinely scheduled follow up appointments [9]. This model incurred costs, in terms of financial and human resources, and did not lead to enhanced detection of recurrent disease [4].

In the past decade, many breast cancer units have transitioned away from the traditional annual doctor-led review towards a more patient-led surveillance programme with responsive access to clinical staff, if required. This is known by a variety of names and will be referred to as “Open Access Follow-Up” (OAFU) in this report. The OAFU programme offers breast cancer patients the opportunity to participate more actively in their own follow up process for five years following the completion of their treatment. Patients are better informed at the outset through small group meetings or telephone consultations and are offered radiological surveillance. Patients have a clear route to access the clinical teams in times of need, but do not have routine clinical review, allowing them to return to “normal life” after breast cancer. This format honours the principles of patient autonomy and self-determination and empowers patients to be in control of their survivorship. An additional benefit is the reduced health care cost, an important factor in many resource-constrained settings and the freeing up of clinical resources to better serve women with concerns or clinical needs in a timely fashion.

However, now that patients no longer routinely attend clinics, the opportunity for patients and doctors to engage and communicate about the long-term effects of treatment is lost. Traditional follow-up allowed surgeons to regularly review their long-term post-operative oncological and aesthetic outcomes which enabled learning and development, ultimately benefiting patients through improved surgical techniques and approaches. It also offered an opportunity for patients to provide treatment-specific feedback and allow their voices to be heard. There is a well-established relationship between patient satisfaction with their post-treatment appearance, psychological well-being, and improved quality of life [[10], [11], [12], [13]].

The use of Patient Reported Outcome Measures (PROMs) is well-established within the breast cancer population [14,15]. Routine collection of PROMs and medical photography in patients undergoing oncoplastic breast surgery is now considered to be standard of care [16,17].

The BREAST-Q questionnaire developed in 2009 by Pusic et al. is a reliable, well-validated [18] tool which evaluates patient satisfaction with appearance and health-related quality of life parameters (physical, psychosocial, and sexual). Specific BREAST-Q modules can be selected according to the type of surgery undertaken e.g., mastectomy, breast reconstruction or breast-conserving surgery. It offers insight into specific patient pathways and satisfaction and large series now provide standards for benchmarking, to highlight areas for improvement and report on effectiveness of technical modifications [14,19,20].

There is a significant volume of literature reporting the utility of oncoplastic multidisciplinary meetings as fora to aid complex decision-making within an oncoplastic team [21,22]. These meetings, however, rarely include post-operative feedback on the outcome of the decisions made and inclusion of these may allow for a team discussion about PROMs and aesthetic outcome results, both good and bad, such that learning opportunities are maximised.

The aim of this study was to evaluate the implementation of a program designed to capture regular patient-reported outcome data and medical photography and to feed it back to the treating team as a group learning opportunity. We specifically sought factors associated with completion of BREAST-Q and attendance at medical photography using two different methods of data acquisition. Secondarily, we report on our patient satisfaction and panel assessment results in routine care.

## 2 Methods

Internal institutional ethics approval was obtained for this evaluation of service (SE850) and funding was provided by an internal innovation grant.

The study consisted of two cohorts of patients. The first cohort of patients were due for their annual surveillance mammogram at either year 3 or year 5 between December 2019 and January 2023. These participants were invited to complete an online version of the BREAST-Q questionnaire using the PROFILES online platform and attend medical photography, coinciding with their follow up mammography appointments. The second cohort consisted of patients who were due their 3- or 5-year surveillance mammography between February 2023 and March 2024 and this group was asked to complete paper BREAST-Q questionnaires. They were also asked to either attend medical photography at the time of their mammogram or to email their “selfies” to a secure hospital medical photography email if preferred. The majority of the participants completed either the 3- or 5-year questionnaires and the few who reached both time points during the duration of the study were invited to complete both sequentially.

Participants who do not attend for annual mammography (because they had a bilateral mastectomy or were young and having MRI surveillance) were excluded (Table 1). Informed consent for participation and medical photography was obtained from the patients.

**Table 1:** Inclusion and exclusion criteria.

Inclusion Criteria	Exclusion Criteria
Patients >18 years of age	Women diagnosed with breast cancer who did not undergo surgical treatment
Have undergone surgical treatment for breast cancer including:	Women not attending annual mammography (bilateral mastectomy)
- Breast conserving surgery (wide local excision, therapeutic mammoplasty) - Simple mastectomy without reconstruction - Mastectomy with immediate reconstruction (implant or autologous)	
Patients in the OAFU Programme	Women who have been removed from the OAFU tracker because of recurrence or out of area
	Patients who received treatment elsewhere and transferred into OAFU

### 2.1 Data collection, storage, and handling

Clinicopathological data was collected from the electronic patient records and entered into a secure password-protected Excel spreadsheet. Patients were photographed by the hospital medical photographers or could send their “selfies” to a secure medical photography email address. Images were securely stored in the electronic patient records, as for routine care. Data collected using the BREAST-Q questionnaires were securely stored online using the PROFILEs directory, an established web-based tool used at that time by the Royal Marsden Hospital for electronic collection of PROMs. It enables participants to complete questionnaires online from any device using their unique login details provided by their hospital team, with all data held at the hospital, ensuring high data security. In January 2023, a decision was made to change to paper-based BREAST-Q questionnaires (cohort 2) given the hospital's plan to decommission PROFILES and the poor response rate to online questionnaires in cohort 1. These paper-based questionnaires were posted to patients with pre-paid return envelopes, collected and stored in a locked office, accessed only by the team members involved in data collection.

BREAST-Q scores from all domains were transformed to a Rasch score on a scale of 0–100 (Q-score) as per the BREAST-Q protocol, with a higher score representing a more favourable outcome [18,23]. Domains such as breast satisfaction, psychosocial, physical, and sexual wellbeing, as well as effects of radiotherapy and satisfaction with staff are common to the questionnaires for all three surgical groups. The breast reconstruction questionnaire includes additional domains such as satisfaction with abdomen, physical wellbeing of the abdomen, satisfaction with implant and nipple reconstruction.

### 2.2 Free text feedback

Participants were given the option to provide free text feedback at the end of the questionnaire by answering the question “If there is anything specific you would like to tell us about your experience to help us achieve excellent results for future patients, please write it here”.

### 2.3 Panel assessment

Remote panel assessment was conducted for both cohorts of patients by six consultant breast surgeons, one consultant plastic surgeon and 3 senior breast surgery fellows, all from the same institution. Assessment was performed in isolation by each panellist, and they were blinded to each other's responses. Panellists were asked to assess the two-dimensional anterior and oblique photographic images using specific scoring systems for the different types of breast surgery. The 4-point Harvard cosmesis score [24] was used for those who underwent BCS (Table 2) and breast reconstruction patients were evaluated using a scale developed through a Delphi consensus process specifically for breast reconstruction following mastectomy (Table 3) [25]. As there is currently no widely adopted scoring system for assessment post simple mastectomy without reconstruction, a reporting system was devised (Table 4). Mean panel scores and Fleiss Kappa was calculated to determine the level of agreement between the panellists for each cohort.

**Table 2:** Harvard cosmesis scoring system [24].

Outcome	Description	Score
Excellent	Treated breast nearly identical to untreated breast	4
Good	Treated breast slightly different from untreated breast	3
Fair	Treated breast clearly different from untreated breast but not seriously distorted	2
Poor	Treated breast seriously distorted	1

**Table 3:** Breast reconstruction scoring system [25].

		Excellent 5	Good 4	Moderate 3	Poor 2	Very poor 1
**Shape**	The overall shape of the reconstructed breast/s	Shape symmetry out of bra achieved	Shape of operated breast is pleasing but not symmetrical	Moderate difference in shape but does not detract from overall aesthetic result	Moderate focal deficits detracting from overall aesthetic result	Large focal deficits distorting contour and significantly detracts from overall aesthetic result
**Volume**	Overall volume symmetry between breasts	Equal volume between breasts	Minor difference in volume	Moderate difference in volume but does not detract from overall aesthetic result	Volume difference impacts overall aesthetic result	Major volume mismatch significantly detracts from overall aesthetic result
**Nipple position**	Nipple position in relation to the ipsilateral breast	Excellent symmetry between sides and nipple in an ideal position on reconstructed breast mound	Minor adjustments required to achieve excellence in nipple position	Noticeably suboptimal but does not influence overall aesthetic result	Nipple position slightly impacts overall aesthetic result	Nipple position significantly detracts from overall aesthetic result
**Projection**	Patient view of symmetry	Projection is equal	Minor differences in projection	Noticeable difference but not detracting from overall aesthetic result	Slightly impacts overall aesthetic result	Significantly detracts from overall aesthetic result
**Position of BreastMound**	In relation to chest wall and other breast	Equal to the other side and in an optimal position on chest wall	Minor asymmetry of position or symmetrical but suboptimal position	Asymmetry of position or symmetrical but suboptimal position not detracting from overall aesthetic result	Slightly impacts overall aesthetic result	Significantly detracts from overall aesthetic result
**Symmetry**	Comparison between breasts	Out of bra symmetry achieved	Mild asymmetry	Moderate asymmetry but does of detract from overall aesthetic result	Moderate asymmetry detracting from overall aesthetic result	Significant asymmetry detracting from overall aesthetic result
**Global**	Taking into consideration subscale evaluation what is your overall impression of the quality of the reconstruction	Excellent	Good	Moderate	Poor	Very poor

**Table 4:** Simple mastectomy scoring system.

Description	Scale
Prominence of scar	1 = Large
Adherence of skin to the chest wall	2 = Moderate
Presence of excess skin, dog ear or swelling	3 = None or minimal
Overall score for appearance	1 = Poor
	2 = Satisfactory
	3 = Good
	4 = Excellent

### 2.4 Data analysis

The data were analysed using SPSS (Version 29) and are presented using descriptive statistics using mean and standard deviation, or median and inter-quartile range, depending on the distribution of the data. Chi square test was used to determine whether uptake was significantly different between the two cohorts. Binary logistic regression analysis was performed to identify clinicopathological variables associated with returning the questionnaire, and with attendance at medical photography for the whole cohort. In order to investigate whether different methods of questionnaire distribution affect uptake in different groups, this analysis was also undertaken for the two cohorts separately. Analysis of BREAST-Q results and panel assessment was undertaken for the study population as a whole since the method of acquisition would not be expected to influence satisfaction with outcome. Similarly, factors associated with attendance at medical photography were also assessed.

One-way ANOVA was used to determine statistically significant differences (p < 0.05) in BREAST-Q scores between the three surgical groups (BCS, mastectomy and reconstruction) and students *t*-test was used to test for statistically significant differences between the two reconstruction groups (implant and autologous).

Free text patient feedback underwent thematic analysis to determine whether consistent themes were present.

A selection of cases for whom both BREAST-Q score and photographs were available were presented at the oncoplastic multidisciplinary meeting on two occasions. Some cases for whom both PROMs and panel assessment were favourable or unfavourable, and some for whom the results were discordant were discussed. Free text feedback, grouped by theme, was also reported to the team.

## 3 Results

The number of women invited, their demographics, and uptake are reported in Table 5. Eleven participants were excluded in cohort 1 because of incorrect or insufficient data, and two patients were excluded as they had not undergone surgery and are under active monitoring within the LORIS study [26] (see Table 6).

**Table 5:** Patient uptake and demographics for cohort 1 and 2 There was a statistically significantly higher questionnaire return in cohort 2 than cohort 1 (Chi square test p < 0.001) but there was no significant difference in attendance at medical photography between the two cohorts (p = 0.290). No patients submitted selfies. Questionnaire uptake was similar in those invited at year 3 and year 5, 15.7 % and 16.3 % respectively (p = 0.269 and therefore analysed together.

	Cohort 1 n (%)	Cohort 2 n (%)	Overall
Dates of recruitment	Dec 2019–Jan 2023	Feb 2023–Mar 2024	Dec 2019–Mar 2024
Missing data	Jan–May 2020	None	Jan–May 2020
	Dec 2021		Dec 2021
Number scheduled for mammography	826	398	1224
Number eligible	813 (98.4 %)	398 (100.0 %)	1211 (98.9 %)
Mean age at study invitation (years) (SD)	62.8 (9.8)	64.0 (10.5)	63.2 (10.1)
Ethnicity			
White British	722 (88.8 %)	344 (86.4 %)	1066 (88.0 %)
Other	91 (11.2 %)	54 (13.6 %)	145 (12.0 %)
Mean BMI (kg/m2) (SD)	27.8 (5.6)	27.5 (5.8)	27.7 (5.7)
Completed BREAST-Q	130 (16.0 %)	116 (29.1 %)	246 (20.3 %)
All photography attendance	116 (14.3 %)	66 (16.5 %)	182 (15.0 %)
Attended medical photography	116 (14.3 %)	66 (16.5 %)	182 (15.0 %)
Self-photography		0 (0.0 %)	0 (0.0 %)
Questionnaire and photographs both available	62 (7.6 %)	52 (13.1 %)	114 (9.4 %)

**Table 6:** Rates of uptake by type of surgery – cohort 1.

Type of surgery	Total number (n = 813)	Questionnaire completed n (%)	Photography attended n (%)	Both n (%)
**Breast-Conserving Surgery (BCS)**	**642 (79.0 %)**	**99 (15.4 %)**	**89 (13.9 %)**	**48 (7.5 %)**
Simple WLE	508 (62.5 %)	72 (14.2 %)	70 (13.8 %)	37 (7.3 %)
Complex oncoplastic procedure	134 (16.5 %)	27 (20.1 %)	19 (14.2 %)	11 (8.2 %)
**Unilateral Simple Mastectomy**	**56 (6.9 %)**	**9 (16.1 %)**	**7 (12.5 %)**	**5 (8.9 %)**
**Unilateral Mastectomy & Recon**	**113 (13.9 %)**	**22 (19.5 %)**	**20 (17.7 %)**	**9 (8.0 %)**
**Unilateral Autologous Recon**	**63 (7.8 %)**	**11 (17.5 %)**	**11 (17.5 %)**	**6 (9.5 %)**
Immediate DIEP	52 (6.4 %)	8 (15.4 %)	7 (13.5 %)	5 (9.6 %)
Delayed DIEP	10 (1.2 %)	3 (30.0 %)	4 (40 %)	1 (10 %)
TRAM	1 (0.1 %)	**--**	**--**	**--**
**Unilateral Implant Recon**	**50 (6.2 %)**	**11 (22 %)**	**9 (18 %)**	**3 (6 %)**
Immediate	49 (6.0 %)	11 (22.5 %)	8 (16.3 %)	3 (6 %)
Delayed	1 (0.1 %)	–	1 (100 %)	–
**Other Surgery**	**2 (0.3 %)**	**--**	**--**	**--**
ALND only	1 (0.1 %)			
Incidental finding	1 (0.1 %)			
**Overall**	**813**	**130/813 (16.0 %)**	**116/813 (14.3 %)**	**62/813 (7.6 %)**

**Table 7:** Rates of uptake by type of surgery – cohort 2.

Type of surgery	Total number (n = 398)	Questionnaire completed n (%)	Photography attended n (%)	Both n (%)
**Breast-Conserving Surgery (BCS)**	**321 (80.7 %)**	**88 (27.4 %)**	**48 (15 %)**	**35 (10.9 %)**
Simple WLE	229 (57.5 %)	52 (22.7 %)	27 (11.8 %)	19 (8.3 %)
Complex oncoplastic procedure	92 (23.1 %)	36 (39.1 %)	21 (23.1 %)	16 (17.6 %)
**Unilateral Simple Mastectomy**	**26 (6.5 %)**	**9 (34.6 %)**	**3 (11.5 %)**	**3 (11.5 %)**
**Unilateral Mastectomy & Recon**	**51 (12.8 %)**	**19 (37.3 %)**	**15 (29.4 %)**	**14 (27.5 %)**
**Unilateral Autologous Recon**	**31 (7.8 %)**	**11 (35.5 %)**	**10 (32.3 %)**	**9 (29.0 %)**
Immediate DIEP	23 (5.8 %)	7 (30.4 %)	6 (26.1 %)	5 (21.7 %)
Delayed DIEP	4 (1.0 %)	3 (75.0 %)	3 (75.0 %)	3 (75.0 %)
Local flap	2 (0.5 %)	0	0	0
TRAM	1 (0.3 %)	1 (100 %)	1 (100 %)	1 (100 %)
LD	1 (0.3 %)	0	0	0
**Unilateral Implant Recon**	**20 (5.0 %)**	**8 (40.0 %)**	**5 (25.0 %)**	**5 (25.0 %)**
Immediate	20 (5.0 %)	8 (40.0 %)	5 (25.0 %)	5 (25.0 %)
Delayed	0	–	–	–
**Overall**	**398**	**116/398 (29.1 %)**	**66/398 (16.6 %)**	**52/398 (13.1 %)**

### 3.1 Factors associated with participation

Binary logistic regression of the whole population of eligible patients revealed that those who had undergone more complex oncoplastic breast conservation procedures were more likely to respond to the questionnaire OR 1.90 (CI 1.33–2.73, p < 0.001) and that those with a higher BMI were less likely to respond OR 0.95 (CI 0.93–0.98, p = 0.001). Being in cohort 2 was found to be an independent factor associated with increased questionnaire response OR 2.16 (CI 1.62–2.88, p < 0.001). Patients who were currently smoking were less likely to attend medical photography OR 0.70 (CI 0.52–0.95, p = 0.023).

Age, ethnicity, type of surgery, axillary procedure, re-excision of margins, complications and elective surgical revisions did not have significant associations with questionnaire or medical photography uptake.

### 3.2 BREAST-Q results

Median BREAST-Q scores for combined cohorts are summarised in Table 8, Table 9. BREAST-Q scores range from 0 to 100, where 0 is the lowest and 100 is the highest, except for satisfaction with implant and abdomen which have a maximum score of 8 and 12 respectively. BREAST-Q results are in line with the literature, with simple mastectomy patients scoring lowest and BCS patients scoring highest for breast satisfaction and autologous reconstruction patients scoring statistically significantly higher for breast satisfaction than implant reconstruction patients.

**Table 8:** Median BREAST-Q results for all respondents.

	Breast-Conserving Surgery (n = 187)	Mastectomy (n = 18)	Reconstruction (n = 41)	P value
**Breast satisfaction**	72 (IQR 54–100)	58 (IQR 44–64)	67 (IQR 52–84)	0.019
**Psychosocial wellbeing**	83 (IQR 64–100)	62 (IQR 52–87)	79 (IQR 61–93)	0.031
**Sexual wellbeing**	62 (IQR 43–84)	27 (IQR 17–55)	59 (IQR 41–66)	0.003
**Physical wellbeing (chest)**	100 (IQR 100-100)	80 (IQR 64–100)	85 (IQR 76–100)	0.537
**Effect of radiotherapy**	100 (IQR 78–100)	100 (IQR 95–100)	87 (IQR 83–100)	0.954

**Table 9:** **Median BREAST-Q results specific to breast reconstruction for all respondents** (satisfaction with abdomen is scored out of a maximum of 12, implant a maximum of 8).

	Implant (n = 19)	Autologous (n = 22)	p value
**Breast satisfaction**	53 (IQR 43–63)	79 (IQR 62–88)	<0.001
**Psychosocial wellbeing**	74 (IQR 51–97)	80 (IQR 65–93)	0.204
**Sexual wellbeing**	58 (IQR 40–62)	59 (IQR 43–68)	0.350
**Physical wellbeing (chest)**	85 (IQR 76–92)	89 (IQR 77–100)	0.065
**Physical wellbeing abdomen**	–	81 (IQR 66–100)	–
**Satisfaction with abdomen**	–	11/12 (IQR 9–12)	–
**Satisfaction with implant**	5/8 (IQR 4–6)	–	–

### 3.3 Free text themes

A total of 137 free text feedback comments were submitted, 72/813 (8.9 %) of cohort 1 and 64/398 (16.1 %) of cohort 2. Thematic analysis identified 12 clear themes, listed in Table 10. Participant feedback was predominantly positive. The most common negative comments were related to information provision, particularly post-operative recovery, and preparation for the likely post-operative appearance. Care during the COVID-19 pandemic was also identified as a theme.

**Table 10:** Thematic analysis of free text feedback.

Theme identified	Number
1. General compliments & compliments related to individual staff members	55
2. Post-operative discomfort/dissatisfaction information provision or support	23
3. Dissatisfaction with pre-treatment information provision, consenting and treatment options (info on surgery, treatment side-effects, recurrence risk, recovery)	14
4. Support and information following treatment completion (OAFU, BCN, consistency of staff)	10
5. Radiotherapy side-effect related	7
6. Dissatisfaction with teams (nurses, doctors, office staff)	7
7. Dissatisfaction with service provision during COVID-19 pandemic	6
8. Dissatisfaction with post-operative appearance	5
9. Dissatisfaction with administration (appointments, contact numbers etc)	3
10. Endocrine treatment side-effect related	3
11. Dissatisfaction with general pre-operative experience (waiting area, change of surgeon)	2
12. Management of post-operative complication	2

### 3.4 Panel assessment

A total of 167 images underwent panel assessment by 10 panellists. The mean global assessment scores are given in Table 11. Agreement between assessors was low, precluding further analysis.

**Table 11:** Panel assessment agreement for overall scores.

	Mean panel score	Agreement (Fleiss Kappa)
**Breast Conserving Surgery** (n = 124)	3.00/4	0.37 – fair
**Simple mastectomy** (n = 11)	2.78/4	0.22 – fair
**Breast Reconstruction – Implant** (n = 10)	3.00/5	0.18 – slight
**Breast Reconstruction – DIEP** (n = 22)	3.99/5	0.22 – fair

## 4 Discussion

This study evaluated a program designed to capture patient-reported outcome measures and aesthetic outcome as part of routine care, and to feed this information back to the clinical team. This approach differs from other reports using breast surgery PROMs and photography in which data are generally given as part of a research study investigating a specific question, rather than to inform group learning as a matter of routine practice.

Although overall questionnaire response and medical photography attendance was low (20.3 % and 15.0 % respectively), the completion of BREAST-Q questionnaires was found to be significantly higher in the second cohort (29.1 %). This could have been due to the return of normality after the COVID pandemic, however, one would have anticipated a greater effect on attendance at medical photography which is, by definition, face to face. As there was a statistically significant improvement in questionnaire response rate and not in medical photography attendance between the two cohorts, we hypothesise that questionnaire completion improved because of participant preference for paper-based questionnaires rather than the online platform. The online platform by which patients accessed the questionnaires was cumbersome, potentially discouraging participants from contributing. While paper questionnaires resulted in an increased response rate, future use of more easily accessible, online questionnaires may lead to further gains. This highlights the importance of making PROMs collection as user-friendly and accessible as possible and ideally offering a number of options for communication and seeking individual patient preference. Clinician-endorsement of this feedback process when patients enter the OAFU programme may also encourage uptake. Contacting patients before sending out the questionnaire and following up with further contact after sending has been shown to increase response rate in research studies [27] but may not be feasible in routine care.

Questionnaire response rates were not statistically significantly different between women invited in year 3 and year 5. This is in contrast to the findings in a study by Santosa et al. which investigated longer-term PROMs reported in participants of the Mastectomy Reconstruction Outcomes Consortium (MROC) study, where the response rate was 21 % at 3 years and only 10.2 % at 4 years [28]. Knowledge that our own patients are equally likely to respond at year 5 as year 3 leads us to plan focus our future data collection at the single, later timepoint.

Age, ethnicity, and surgical factors (type of surgery, axillary procedure, re-excision of margins, post-op complications and elective revisional surgery) were not associated with response to the questionnaire or medical photography attendance. Patients with a higher BMI and current smokers were less likely to participate in the study. This may be because these factors may result in poorer aesthetic outcome and lower patient satisfaction with breasts, as demonstrated in previous studies [14,29,30]. Participants who underwent more complex oncoplastic procedures (e.g., mammoplasty) were more likely to respond to the questionnaire than those who had a simple wide local excision, possibly indicating greater satisfaction with their post-operative aesthetic appearance or greater connection with the surgical team.

BREAST-Q response and attendance at medical photography were proportionally highest in the breast reconstruction group (25.0 % of reconstruction patients participated) which may reflect enhanced patient engagement with the treating team, given that patients undergoing breast reconstruction tend to have more frequent pre-and post-surgery follow up appointments and clinician interactions with the surgical team.

Medical photography attendance was proportionally lowest in the simple mastectomy group (12.2 % of eligible simple mastectomy patients participated), which may reflect lesser patient satisfaction with their aesthetic result or the fact that this patient group may not be as familiar with medical photography as patients undergoing oncoplastic or reconstructive surgery who have generally had baseline photographs and seen others’ photographs as part of their surgical decision-making. Further research into understanding study engagement in women who undergo simple mastectomy is warranted.

BREAST-Q results were generally in keeping with existing literature [[31], [32], [33], [34], [35], [36]] with simple mastectomy patients scoring lowest for breast satisfaction, psychosocial-, sexual- and physical wellbeing. This work provided a learning opportunity not only for trainees but also for our oncoplastic breast surgery team as a whole.

Results including free text feedback were presented to the clinical teams and found to be useful. Free text feedback highlighted areas of concern for patients and provided us with useful insights, such as the need for better information about likely post-operative appearance, which can help future patients make informed choices. An insight unique to cohort 2 was the impact of the COVID-19 pandemic on patient anxiety surrounding their care at that time and a number of patients felt that the reduced face to face clinician interaction at the time negatively impacted their treatment experience.

Agreement between panel assessment scores was low across all surgical groups. This is consistent with the literature which has shown that panel assessment, although the most widely accepted technique of post-operative aesthetic assessment, lacks repeatability, reliability, and interpretability [37,38]. Further development and potential automation of existing aesthetic outcome models [38,39] would lead to efficient, objective assessment of post-operative outcome without the shortfalls of panel assessment. While this is available for breast-conserving treatment [40] it is not yet possible for mastectomy patients.

Over 1000 women were invited to participate in this evaluation. Although there were a number of possible reasons for low uptake in cohort 1, we responded to this by offering an alternative method to complete the questionnaire and to submit photographs. This appears to have improved questionnaire completion but made little difference to photography. We continue to strive to improve this. Another strength is the relatively long-term patient-reported outcomes and panel assessment following breast surgery. We intend to introduce the concept of medium-term evaluation to patients at the end of hospital-based treatment, and to offer women the opportunity to state their preferred method of questionnaire submission and medical photography to make this more user-friendly, which we hope will result in further improved uptake, and more complete and less biased feedback to the team. This is also the first study to formally assess the aesthetic appearance following simple mastectomy using a scoring system. There are numerous existing scoring systems for the appearance following BCS(24) and breast reconstruction [25,41], but not following simple mastectomy.

This study has some limitations. It reports on a single site of a single centre and the reasons for low uptake may not be widely generalisable. Nonetheless, there are plausible explanations, some of which have been addressed. As for all retrospective studies of PROMs, this evaluation may be affected by sampling bias as patients who are very satisfied or dissatisfied may be more likely to respond than those who feel ambivalent [27]. While scoring of contemporary images and the questionnaire domains on current satisfaction with the breasts and other quality of life metrics are not subject to recall bias, the retrospective elements such as satisfaction with peri-operative information provision are vulnerable.

## 5 Conclusion

This study highlights some of the issues involved in routine capture and processing of PROMs and aesthetic outcome data. Breast units must work hard to be responsive to their local demographic and to encourage sharing of this information by patients. Our team found the review of outcome data informative.

## Ethical approval

Internal institutional approval was obtained for this evaluation of service since this represents standard of care.

## Conflict of interest

None.

## CRediT authorship contribution statement

**Astrid E. Leusink:** Writing – original draft, Project administration, Investigation, Data curation. **Amy R. Godden:** Writing – review & editing, Project administration, Funding acquisition, Data curation. **Nihal Yildirim:** Writing – review & editing, Investigation, Data curation. **Antonia Randawa:** Data curation. **Rebekah Law:** Writing – review & editing, Data curation. **Jennifer E. Rusby:** Writing – review & editing, Visualization, Supervision, Methodology, Investigation, Formal analysis, Conceptualization.
